# The Interplay between QSAR/QSPR Studies and Partial Order Ranking and Formal Concept Analyses

**DOI:** 10.3390/ijms10041628

**Published:** 2009-04-17

**Authors:** Lars Carlsen

**Affiliations:** 1 Awareness Center, Hyldeholm 4, Veddelev, DK-4000 Roskilde, Denmark; E-Mail: LC@AwarenessCenter.dk; Tel. +45 2048 0213; Fax: +45 2049 75786; 2 Department of General and Applied Chemistry, Kazakh-British Technological University, Tole Bi str. 59, Almaty 050000, Kazakhstan

**Keywords:** QSAR, QSPR, Partial Order Ranking, Hasse diagrams, Posets, Formal Concept Analysis, Rocket fuel, Environmental impacts, Human health impact, Risk assessment

## Abstract

The often observed scarcity of physical-chemical and well as toxicological data hampers the assessment of potentially hazardous chemicals released to the environment. In such cases Quantitative Structure-Activity Relationships/Quantitative Structure-Property Relationships (QSAR/QSPR) constitute an obvious alternative for rapidly, effectively and inexpensively generatng missing experimental values. However, typically further treatment of the data appears necessary, *e.g.*, to elucidate the possible relations between the single compounds as well as implications and associations between the various parameters used for the combined characterization of the compounds under investigation. In the present paper the application of QSAR/QSPR in combination with Partial Order Ranking (POR) methodologies will be reviewed and new aspects using Formal Concept Analysis (FCA) will be introduced. Where POR constitutes an attractive method for, *e.g.*, prioritizing a series of chemical substances based on a simultaneous inclusion of a range of parameters, FCA gives important information on the implications associations between the parameters. The combined approach thus constitutes an attractive method to a preliminary assessment of the impact on environmental and human health by primary pollutants or possibly by a primary pollutant well as a possible suite of transformation subsequent products that may be both persistent in and bioaccumulating and toxic. The present review focus on the environmental – and human health impact by residuals of the rocket fuel 1,1-dimethylhydrazine (heptyl) and its transformation products as an illustrative example.

## 1. Introduction

In recent years there has been an increasing focus on the possible negative effects to the environment and to the human health from xenobiotics accidentally or deliberately released into our environment. Consequently, the assessment and regulation of chemicals has over the years developed to a major issue in relation to assuring the human health as well as to protect our environment. However, due to an apparent significant lack – or unavailability – of both physico-chemical and toxicological data the vast majority of the chemicals available on the market today has not been properly assessed and regulated. Further, a comprehensive assessment may in many cases be hampered by the fact that only the primary pollutant is assessed whereas the possible multitude of potentially hazardous transformation products escape the assessment simply due to the lack of data. For a discussion of data availability see, *e.g.*, [1–3].

Deriving data based on Quantitative Structure-Activity Relationships/Quantitative Structure-Property Relationships constitutes as an attractive supplement or even alternative to an experimental data generation, the latter being both time consuming and costly.

In the present review the application of QSAR/QSPR methodologies to investigation the environmental and human health impact of residual rocket fuel, 1,1-dimethylhydrazine (**1**), as well as a series of its transformation products will be used as an illustrative example [4–6]. This example further constitutes an illustration of the above mentioned problem associated with primary and secondary pollutants.

Although applying a suite of appropriate QSAR/QSPR models will lead to the required data for the single substances, a further analysis of the mutual relations between the single substances under investigation may appear appropriate. Partial order ranking methodologies appear in this connection as a highly attractive point of departure as this method allows mutual ranking of, *e.g.*, a series of chemical substances based on a simultaneous inclusion of several parameters, like, *e.g.*, persistence, bioaccumulation and toxicity [7,8]. In the present paper the mutual ranking of 1,1-dimethylhydrazine (**1**) and its transformation products simultaneously based on calculated probabilities for being carcinogenic, mutagenic, teratogenic and embryotoxic will illustrate the principle.

In a proper assessment of the chemical substance, not only the physico-chemical and toxicological characteristics, as the PBT characteristics should be taken into account. Also a series of additional factors may advantageously be considered. Thus, parameters like production tonnage [9], specific release scenarios [9,10], and geographical and site-specific factors in addition to various substance dependent parameters should be taken into account. Further socio-economic factors may be taken into consideration as being illustrated in a series of previous papers [11–15]. The more elaborate hierarchical partial order ranking (HPOR) [16] where a larger variety of parameters, *e.g.* originating from various sources and subsequently combined are taken into account have been applied to give a more comprehensive picture of the human health impact originating from a possible exposure to residual rocket fuel and its transformation products.

To further uncover possible linkages among objects and the describing parameters and thus disclose possible synergisms or antagonisms of the parameters formal concept analysis (FCA) [17,18] appears as the appropriate method. The methodology is closely linked to partial order theory and will in this review be illustrated in a further study on the environmental and human toxicological effects of rocket fuel transformation products.

## 2. Results and Discussion

The obvious lack of data when talking about the assessment and eventually the regulation of compounds hazardous to the environment and/or to man unequivocally constitutes an incentive to look for alternative and more rapid ways to obtain the required data. A further incentive to look for alternatives to the conventional experimental methods would be the possibility of reducing the consumption of experimental animals. Classification of chemical compounds based on test involving experimental animals typical requires a significant number of animals for each compound. A reduction in the use of experimental animals is strongly desirable.

Apparently, the main problem to be faced apparently is the dilemma between the fact that decisions must be made, the necessary data to do so, however, are lacking. Theoretically based methods turn up as an obvious possibility. Thus, an attractive alternative appears to be the application of Quantitative Structure and Quantitative Property Activity Relationships (QSAR/QSPR) models for deriving data that may substitute for the lack of experimental data, the basic concept being that molecules that are structurally closely related will display similar properties. This is expressed as the ‘Similar Property Principle’ stating that *‘Structurally similar molecules will exhibit similar physicochemical and biological properties*’ [19].

Since as early as around 1860 a number of researchers [20–23] have applied the inherent notations of the QSAR concept. However, the fatherhood of the QSAR concept as applied today can be ascribed to Hansch [24] through his epoch-making since the beginning of the 50’ties.

Today a major field for application of QSARs is within the field of drug design [25–28]. The application of QSAR techniques enables researchers to screen a significant number of potential drug candidates within a rather short time. Thus, the economic benefits are overwhelming.

Within the last 15–20 years the application of QSAR/QSPR in environmental science has increased [29–33]. Thus, a wide variety of QSAR/QSPR has been developed to predict environmentally crucial physico-chemical parameters such as solubility, distribution, partition, sorption and bioaccumulation as well as ecotoxicological properties (endpoints) [33]. However, also modeling associated to human health, *i.e.* toxicological endpoints has been further developed and QSAR/QSPR with a high predicting power in these areas now are available [33].

Hence, today a wide variety of QSAR/QPSR models are available [33], the vast majority of these being available on a commercial basis only. However, free models of high quality are available. The QSAR/QSPR data derived for the studies covered by the present review are obtained using such models as the EPI Suite from the USEPA [34] for the prediction of physico-chemical parameters as well as ecotoxicological data and the PASS software from the Academy of Medical Sciences, Moscow [35] and the ADME/Tox WEB from Pharma Algoritms [36], the latter being a free web version of the commercially available ADME Boxes and ToxBoxes. In the following data derived from our recent studies on residual rocket fuel, 1,1-dimethylhydrazine (**1**) and a series of its transformation products serve as an illustrative example [4–6].

### 2.1. Residual Rocket Fuel and its Transformation products

The Baikonur Cosmodrome in Kazakhstan has over the years been an important site for rocket launching with more than two thousand launches of different rocket-carriers up to now. Today heavy equipment to the International Space Station (ISS) is transported by ‘Proton’ carriers, the propellant used for these rockets being unsymmetrical 1,1-dimethylhydrazine (**1**), also known as “heptyl”.

The area northeast of the Cosmodrome functions as dropping zone for burned-out rocket fuel containers of the first rocket stage separated in a height of 50 and 100 km (Proton carriers). The fuel containers at this point still contains approx. 0.6 to 4 tons of unburned **1** and about 4 tons of nitrogen oxidants [37]. Significant amounts of residual rocket fuel reach the ground, the actual amount being dependent of the season and are subsequently spread over the surface, where it either evaporates and/or penetrates into the soil [37,38]. Hence, it has been estimated that significant amounts of unburned fuel are being spread over several square kilometers of land.

**Figure 1.**
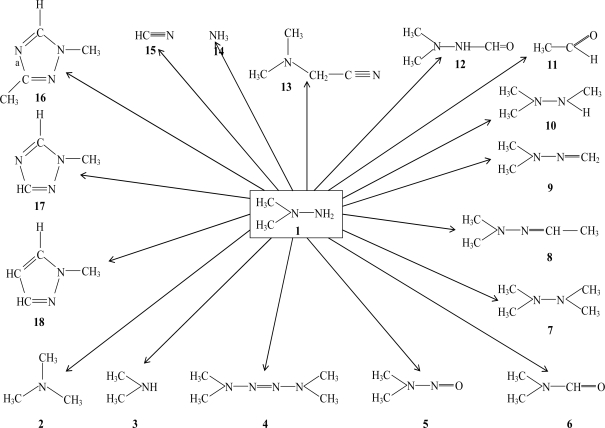
Transformation of 1,1-dimethylhydrazine in soil and water [5,6].

In addition to the pollution with the primary pollutant **1**, a series of so-called secondary pollutants being developed in soil samples polluted by **1** has recently been disclosed [5,6]. This group of compounds constitutes both transformation products that are formed directly from **1** as well as compounds that are formed in various consecutive and possibly surface catalyzed processes. In Figure 1 the the major transformation products disclosed are summarized.

### 2.2. Environmental Behavior of Rocket Fuel and its Transformation Products

In recent year there has been a special focus on compounds being persistent, bioaccumulating and toxic (PBT’s) or very persistent and very bioaccumulating (vPvB’s) [39], as such compounds obviously are of major environmental concern. Further, hazard properties for bulk chemicals are typically linked to the physical-chemical properties such as molecular weight, aqueous solubility, Henry Law constant, vapor pressure, and octanol-water partition constant and the biodegradation probability [40]. In Table 1 a selection of EPI Suite derived physico-chemical data for the **1** and its transformation products is given together with experimental data when available. The good agreement calculated and experimentally obtained values was found noted [5].

Rather high water solubility, log *S_W_*, and correspondingly low octanol-water partition coefficients, log *K_OW_* were found and not surprisingly low to very low Henry Law Constants, log *HLC* for all substances. A high migration potential for these substances was further substantiated through low water-organic carbon partition, log *K_OC_* [5].

The majority of the compounds possess acid-base characteristics that may cause a strong affinity to mineral soil particles and thus less susceptible for biodegradation. Thus, Adushkin [41] found **1** to be very persistent in dry soils, suggesting a self-remediation period of certain soils from **1** of about 34 years.

The relatively high vapor pressures, log *VP*, found [4,5] were associated with an only limited evaporation from an aqueous phase, whereas evaporation from top layers of dry soils could be significant thus reducing a possible terrestrial pollution. In addition also biodegradation should be taken into account. From Table 2 is seen that all compounds apparently rapidly are degraded, the ultimate biodegradation half lives being within weeks, apart from **5** and **13**. Furthermore half of the compounds were predicted to be anaerobically degradable. In Table 2 further the calculated residence times in “standard” rivers and lakes [5] (cf. Section 3.1.2) are collected.

A deeper discussion on the implications of the above figures is outside the scope of the present review and the reader is advised to consult the original papers by Carlsen *et al*. [4,5]. However, for completeness it should be mentioned that none of the compounds possess any significant bioaccumulation potential.

### 2.3. Ecotoxicology of Rocket Fuel and its Transformation Products

The environmental toxicity of the compounds were derived [4,5] applying the ECOSAR module of the EPI Suite leading to non-polar base line toxicity and polar acute toxicities towards fish, daphnids and green algae as summarized Table 3. Further the chronic toxicities and in certain cases the toxicities towards earthworms were predicted (data not shown here) [5].

From the figures given in Table 3 Carlsen *et al*. {5] concluded that apart from the primary pollutant, **1**, and for the compounds **7** – **10** the investigated compounds apparently will not constitute any significant toxicity towards neither aquatic nor terrestrial organisms.

**Figure 2.**
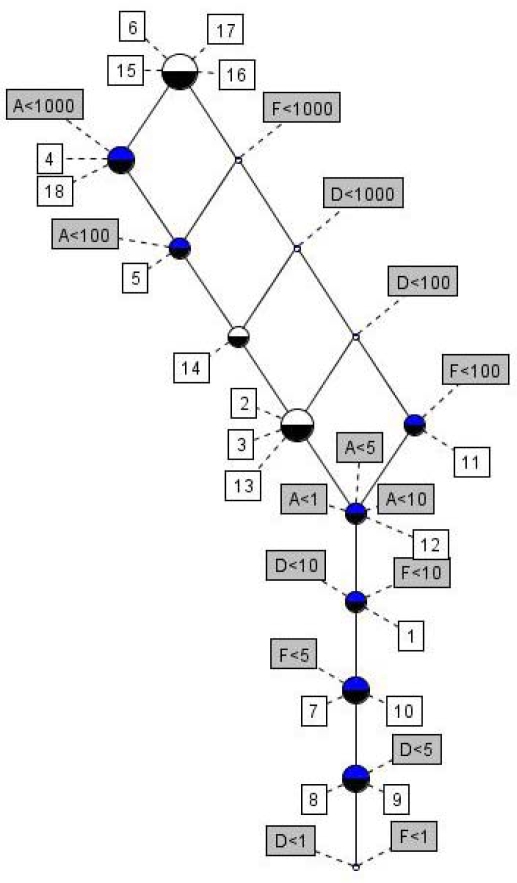
Lattice line diagram for ecotoxicological effects by 1,1-dimethylhydrazine and its transformation products as derived by EcoSAR [46].

To further analyze the above data for acute toxicity a formal concept analysis was conducted in order possibly to reveal possible synergisms or antagonisms with the group of compounds [42]. In Figure 2 is displayed the line lattice diagram for ecotoxicological effects by **1** and its transformation products as derived by EcoSAR, the behind lying context table being given as Appendix 1.

Obviously the diagram contains a significant number of trivial information, like if the toxicity towards fish for a given compound < 1 mg/L it is also < 10, 100 and 1000 mg/L, respectively. However in addition to such information a series of implication sets and association rules pointed to the fact that for several of the compounds in the study toxicological effects on several species prevailed. Thus, from the FCA it was concluded [42] that for 7 compounds displaying acute toxicities towards fish at concentrations below 100 mg/L (F < 100) also displayed toxicities to daphnids below 100 mg/L (D < 100). Likewise implications were disclosed that for 5 compounds with F < 10 mg/L then D < 10 and A < 1 mg/L, for 4 compounds with F < 5 mg/L then D < 10 and A < 1 mg/L and for 5 compounds with D < 10 mg/L then F < 10 and A < 1 mg/L, respectively.

Further it was disclosed that for six out of seven compounds (86%) with D < 100 mg/L then A < 1 mg/L, for five out of six compounds (83%) with A < 1 mg/L then F < 10 and D < 10 mg/L and for four out of five compounds (80%) with A < 1, F < 10, and D < 10 mg/L then F < 5 mg/L, respectively [42].

### 2.4. Human Health Impact by Rocket Fuel and its Transformation Products

In a further study Carlsen *et al*. [6] investigated the possible human health impact of **1** and its transformation products (cf. Figure 1). Thus, the probabilities for the substances to carcinogenic, mutagenic, teratogenic and/or embryotoxic were elucidated using the QSAR/QSPR software PASS (Prediction of Activity Spectra for Substances) [35], whereas absorption, distribution, metabolism and excretion (ADME) characteristics and toxicology, *e.g.*, the probabilities for adverse organ specific health effects were disclosed using the ADME Boxes and ToxBoxes [36].

In Table 4 the results of the ADME calculations are shown. It should be noted that neither an active absorption nor any significant 1^st^ pass metabolism was noted for the compounds apart from **13** [6]. For a detailed discussion of the data the original study by Carlsen *et al* [6] should be consulted as it is outside the scope of the present review.

In the study by Carlsen *et al*. [6] also predicted acute toxicities of the rocket fuel and its transformation products (cf. Figure 1) were calculated (data not shown; the original reference should be consulted [6]) are compared to available experimental data.

Carlsen *et al*. [6] found that in some cases, *e.g.*, in the case of **6** the predicted acute toxicities are significant overestimated, whereas in other cases, like **1** and **15** ToxBoxes apparently underestimates the toxicities. In other cases the agreement was found to be acceptable. For a more elaborate discussion the original reference [6] should be consulted.

Based on the above ADME results it was concluded [6] that the compounds apparently would move freely throughout the body and thus travelling in and out of tissues the compounds may perpetrate its biological effects. Based on calculations applying ToxBoxes (Pharma Algorithms1) the probabilities for adverse organ specific health effects (the blood, the cardiovascular and gastrointestinal systems, the kidneys, the liver and the lungs) were elucidated (Table 5). Based on these data it was concluded [6] that the most likely adverse effects are typically predicted to be in the gastrointestinal system.

**Figure 3.**
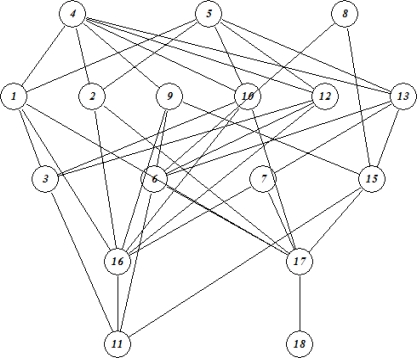
Hasse diagram constructed based on the parameters GAS, LIV and LUN [6].

Based on the above data (Table 5) the overall assessment of the adverse organ specific health effects immediately turns into a multicriteria problem as several parameters simultaneously had to be taken into account. Hence, Carlsen *et al* [6] advantageously applied partial order ranking [11–13,44] for the subsequent data analyses as this method allows simultaneous inclusion of several parameters. In Figure 3 the Hasse diagram constructed based on predicted adverse organ specific health effects, as derived from the ToxBoxes [36] including the gastrointestinal system (GAS), the liver (LIV) and the lungs (LUN), respectively, the more hazardous compounds being located in the top of the diagram. Thus, on a cumulative basis it was concluded [6] that compounds **4**, **5** and **8** were those of major concern followed by the compounds (level 2) **1**, **2**, **9**, **10**, **12** and **13**. The less hazardous compounds, **11** and **18**, are found in the bottom of the diagram.

Carlsen *et al*. [6] further screened the 18 compounds (cf. Figure 1) for possible adverse biological effects applying the web version of the PASS software (PASS1) with the specific focus at carcinogenicity, mutagenicity, teratogenicity and embryotoxicity. In Table 6 the predicted probabilities for the studied substances being carcinogenic, mutagenic, teratogenic and embryotoxic, respectively are summarized. Only probabilities higher than 0.5 were considered.

**Figure 4.**
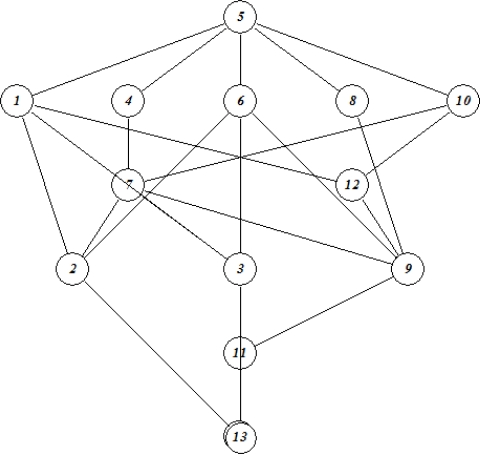
Hasse diagram constructed based on the parameters CAR, MUT, TER and EMB [6]. For calculation purposes probabilities < 0.5 (denoted NE in Table 6) are for ranking purposes arbitrarily set to 0.25 [6].

Analogously to the above ranking of the compounds based on the adverse organ specific effects the compounds were subsequently ranked according to their probabilities ofbeing carcinogenic (CAR), mutagenic (MUT), teratogenic (TER) and embryotoxic (EMB), respectively (Figure 4).

Comparing the two figures (Figures 3 and 4) obviously some differences prevail although a series of the same compounds appear in the top levels of the diagrams. Thus, based on the PASS predictions Carlsen *et al* [6] found that **5** appeared as the most dangerous substances followed by the compounds (level 2) **1**, **4**, **6**, **8** and **10**, respectively. The compounds **13** and **15** – **18** are found in the bottom of the diagram as equivalent elements in agreement with the fact that these compounds all displayed probabilities less than 0.5 for the parameters studied (cf. Table 6).

Since the compounds at the same level in the diagram cannot immediately be compared Carlsen *et al*. [6] calculated the averaged rank of the suite of compounds studied using eqn. 4 (see Section 3) resulting in a linear rank of all compounds. In Table 7 the calculated averaged rank of the 17 compounds based on a) GAS, LIV and LUN and b) CAR, MUT, TER and EMB, respectively are given. Obviously, compounds located in the top level (level 1) in the Hasse diagrams (Figures 3 and 4) are calculated to have the top averaged ranks followed by the compounds found at the subsequent levels in the diagrams.

**Figure 5.**
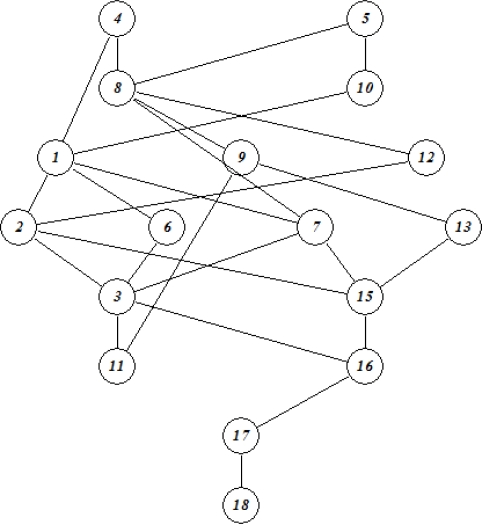
Hasse diagram constructed based on the meta descriptors originating from the ToxBoxes and the PASS calculations, respectively, cf. Table 7 [6].

Subsequently an overall assessment of the human health impact by the rocket fuel **1** and its transformation products was estimated applying the Hierarchical Partial Order Ranking (HPOR) approach [6,16]. Hence, the averaged ranks given in Table 7 were adopted as so-called metaparameters [16] denoting the predicted impact according to the ToxBoxes and the PASS calculations, respectively. Subsequently a further Hasse diagram using these meta-descriptors was constructed (Figure 5) the eventual averaged rank elucidating the overall assessment of the 17 compounds with respect to their adverse human health effects are displayed in Table 8.

From Figure 5 and Table 8 Carlsen *et al*. [6] concluded that in addition to compounds **5** and **4,** the major risk apparently would be associated with the hydrazines and the hydrazine derivatives, **1**, **8**, **9,10**, and **12**. This conclusion appeared to be parallel to the one drawn looking at the possible environmental impact (*vide supra*) apart from the fact that the tetrazene, **4**, apparently does not appear to exhibit major risk in relation to environmental impact [5].

The here presented results (Figure 4 and 5 and Table 7 and 8) are a nice illustration of the usefulness of partial ordering methodologies in attempts to carry out assessments of, *e.g.*, a group of xenobiotics or as studied by Carlsen *et al*. [6] of a group of substances consisting of a primary pollutant and a series of transformation products. Hence, through this assessment it was clearly demonstrated that some of the transformation products could lead to adverse health effects at the same or even higher level than the primary pollutant.

**Figure 6.**
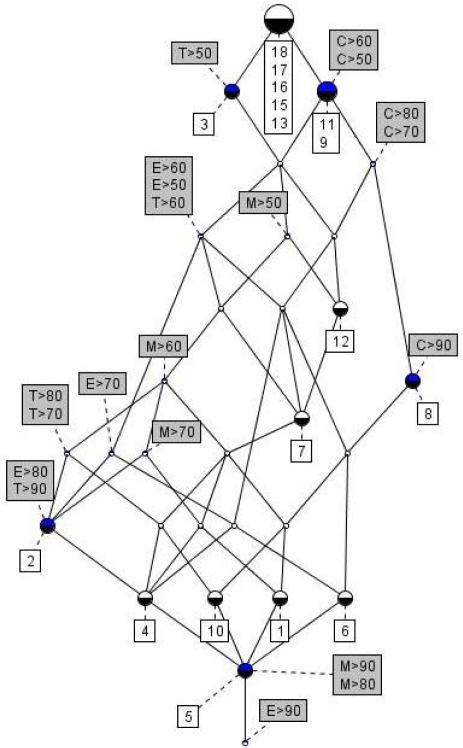
Lattice line diagram for human health effects by 1,1-dimethyl hydrazine and its transformation products as derived by PASS [35].

To further analyze the above data for adverse human health effects a formal concept analysis was conducted in order possibly to reveal possible synergisms or antagonisms with the group of compounds [42]. In Figure 6 is displayed the line lattice diagram for the probabilities of **1** and its transformation products being CAR, MUT, TER and EMB, respectively as derived by PASS, the behind lying context table being given as Appendix 2.

As in the case of the ecotoxicological data also here the diagram display a series of trivial information like, *e.g.* for compounds **5** the probability of being carcinogenic > 90 % (C > 90) it is of course also higher that 80 (C > 80), 70 (C > 70), 60 (C > 60), and 50 % (C >50), respectively.

However, in addition to this trivial information a series of implication sets and association rules pointed to the fact that for several of the compounds in the study a multitude of adverse human health effects prevail. In Tables 9 and 10 selected implications sets and association rules are summarized [42]. The notation like M > 70 denotes that the probability of the compounds to be mutagenic being higher that 70%. C, M, T, and E denoted carcinogenicity, mutagenicity, teratogenicity and embryotoxicity, respectively.

Although the above presented FCA studies include only a limited number of substances it nicely illustrates the possibilities to combine QSAR/QSPR generated data with formal concept analyses and thus retrieving important comprehensive information concerning the possible multitude of effects of a group of compounds.

## 3. Methodology

The basic methodology applied for assessing chemical substances is partial order ranking and formal concept analyses based on QSAR/QSPR generated data. Thus, in the following a description of the applied QSAR/QSPR models will be given. The basic concepts of partial order ranking (POR), including deriving linear extensions (LE), ranking probability and averaged ranks are summarized. Further the more elaborate partial order ranking methodologies, *i.e.*, hierarchical partial order ranking (HPOR) and accumulating partial order ranking (APOR) are described as is the principles and ideas about formal concept analyses (FCA).

### 3.1. Quantitative Structure-Activity/Property Relationships (QSAR/QSPR)

QSAR/QSPR modeling can in the simplest form be expressed as the development of correlations between a given physico-chemical property or biological activity (endpoint), *P*, and a set of parameters (descriptors), *D_i_*, that are inherent characteristics for the compounds under investigation

(1) $$P\;=\;f(D_{i})$$

The properties (endpoints), *P* that has been subjected to QSAR/QSPR modeling comprises physicochemical properties and biological activities in the environment as well in the human beings.

In general models that describe/calculate key properties of chemical compounds take into account three types of inherent characteristics of the molecule, *i.e.*, structural, electronic and hydrophobic characteristics. Depending on the actual model few or many of these descriptors may be taken into account. Thus, eqn. 1 can be rewritten as

(2) $$P\;=\;f(D_{\text{structural},\;}D_{\text{electronic},}\;D_{\text{hydrophobic},}\;D_{x})\;+\;e$$

The descriptors reflecting structural characteristics may, *e.g.*, be element of the actual composition and 3-dimensional conFiguration of the molecule, whereas descriptors reflecting the electronic characteristics may, *e.g.*, be HOMO/LUMO energies, charge densities, dipole moment etc. The descriptors reflecting the hydrophobic characteristics are related to the distribution of the compound between a biological, hydrophobic phase, and an aqueous phase. A further, fourth type of characteristics, *D_x_*, (cf. eqn. 2) accounts for possible underlying characteristics that may be known or unknown, such as environmental or experimental parameters as, *e.g.*, temperature, salt content etc. The data may often be associated with a certain amount of systematic and non-quantifiable variability in combination with uncertainties. These unknown variations are expressed as “noise”. Thus, the parameter, *e*, account for possible noise in the system, *i.e.*, the variation in the property that cannot be explained by the model.

In the studies presented in the present review paper a series of freely available QSAR/QSPR models has been applied. Thus, physico-chemical data, environmental persistence and environmental toxicities have been obtained applying the EPI Suite [32]. The interaction with the human organism has been elucidated through absorption, distribution, metabolism and excretion data derived by ADME Boxes [36] and the human toxicological effects by ToxBoxes [36] and by PASS (Prediction of Activity Spectra for Substances) [35].

### 3.1.1. Physico-chemical data

The EPI Suite has been applied as the primary tool for generating physico-chemical endpoints [34]. This software package includes a variety of submodules to estimate, *e.g.*, water solubility (log *S_W_*) calculated by the submodule WSKOW, octanol-water partition (log *K_OW_*) calculated by the submodule KOWWIN, vapor pressure (log *VP*) calculated by the submodule MPBPWIN, and Henry’s Law constants (log *HLC*) calculated by the submodule HENRY. Sorption to organic carbon was calculated using the submodule PCKOCWIN. The log *K_OW_* values generated in this way are subsequently used to generate bioconcentration factors (log *BCF*) [43] calculated by the submodule BCF program. Substances with log *BCF* < 3.0 were regarded as non-bioaccumulating. Substances exhibiting log *BCF* values of > 3.0, but < 3.70 are assigned a medium bioconcentration potential whereas substances with log *BCF* > 3.70 were assigned a high bioconcentration potential. [34].

#### 3.1.2. Environmental persistence

Through the BioWin module [34] persistence predictions were obtained. The submodule BDP3 provides estimates of a substance’s environmental biodegradation rate by calculating the degradation probabilities. The lower the probability the higher the persistence. Eventually BDP3 returns the biodegradation potential as hours, hours to days, days, days to weeks, weeks, weeks to months and months, respectively, depending on the approximate amount of time needed for a “complete” biodegradation [34,45].

**Table:** 

BDP3	Predicted Half-Lives (days)
Hours	0.17
Hours to Days	1.25
Days	2.33
Days to Weeks	8.67
Weeks	15
Weeks to Months	37.5
Months	60
Recalcitrant	180

Substances with half lives >180 days are assigned high persistence potential, the corresponding BDP3 value being <1.75, whereas substances a half-life in the predominant compartment of ≥ 60 and ≤ 180 days are assigned medium persistence potential, the corresponding BDP3 value being > 1.75 and < 2.0 [45].

The fate in the aquatic media is, in addition to the biodegradation estimated as the potential for volatilization from water. In the present study volatilization from rivers (water depth 1m, wind velocity 5 m/s and current velocity 1 m/s) and from lakes (water depth 1m, wind velocity 0.5 m/s and current velocity 0.05 m/s) was calculated using the WVOLWin module in EPI Suite [34].

#### 3.1.3. Environmental toxicity

Toxicities of the investigated substances have been obtained using the ECOSAR [46] that calculates the toxicity of chemicals discharged into water. Both acute (short-term) toxicities and chronic (longterm or delayed) toxicities are calculated by ECOSAR, the calculations being based on the octanolwater partition (log *K_OW_*). ECOSAR can run independently or as an integrated part of the EPI Suite

ECOSAR return the acute as well as chronic toxicities of the substance under investigation to fish (both fresh and saltwater), water fleas (daphnids), and green algae. In some cases also other effects, *e.g.*, toxicity to earthworms are returned. The acute toxicities are calculated as LC50 values.

#### 3.1.4. Absorption, Distribution, Metabolism and Excretion (ADME)

Predictions for the absorption, distribution, metabolism and excretion (ADME) and Toxicology are obtained using freely and commercially available *in silico* expert systems, *i.e.*, the web version of the ADME Boxes software [36] based on ADME Boxes ver. 3.5. ADME Boxes is modulized software that allows calculation of selected physico-chemical data, oral bioavailability (human), human intestinal absorption, transport, distribution including volume of distribution and plasma bound fraction based on the chemical structure. The software modules are based on exacting data analyses and expert models for calculating the vital properties.

Calculations on the concentration of the single compounds in the plasma as a function of time are generated using the ADME Boxes ver. 4.1 [47] as this feature is currently not implemented in the free web version.

#### 3.1.5. ToxBoxes

Acute toxicity towards mouse and rat as well as the probability of adverse organ specific health effects affecting the blood, the cardiovascular- and gastrointestinal systems, the kidneys, the liver and the lungs, respectively and a positive response in an Ames test is derived using the web version of the ToxBoxes software [36] based on ToxBoxes ver. 2.0. ToxBoxes is modulized software that allows calculation of toxic effects of molecules solely from the chemical structure in combination with expertise in organic chemistry and toxicology.

The validation of the ADME Boxes and ToxBoxes software has been carried out as a validation of the single modules. Overall it can be stated that the accuracy of the ADME Boxes and the ToxBoxes are high. Thus, in the case of Ames test the accuracy was found to be in the order of 95% based on a validation set of ca. 1,700 substances [48]. Typical values for the various modules comparing experimental and predicted values for a series of compounds not being involved in the model development (validation set) were R^2^ higher than 0.8.

#### 3.1.6. Prediction of Activity Spectra for Substances (PASS)

The computer program PASS (Prediction of Activity Spectra for Substances) developed by the Academy of Medical Sciences, Moscow, predicts the biological activity for a compound on the basis of its structural formula [35].

The freely available internet version of PASS allows the prediction of 2,468 pharmacological effects as well as mechanisms of action [49]. For the studies referred to in this review PASS has been used to derive probabilities for the invested compounds to carcinogenic, mutagenic, teratogenic and embryotoxic. In the case of carcinogenicity the highest value predicted (male/female mice, male/female rats) were applied. The PASS training set includes approx. 46,000 biologically active compounds, comprising about 16,000 already launched drugs and 30,000 drug-candidates currently under clinical or advanced preclinical testing. [50]. The accuracy of the PASS predictions has been reported to be approx. 86% [51,52], Thus the maximum error of prediction has been estimation to be approx. 15, 13, 21 and 20% for prediction of carcinogenicity, mutagenicity, teratogenicity and embryotoxicity, respectively [51]. For all compounds referred to in present review, rocket fuel and transformation products, the number of new descriptors are 0, 1 or, at a maximum, 2, respectively, and thus complying with the limitations of the method [53].

### 3.2. Partial Order Ranking (POR)

The theory of partial order ranking is presented elsewhere [44,54]. In brief, Partial Order Ranking is a simple principle, which a priori includes “≤” as the only mathematical relation. If a system is considered, which can be described by a series of descriptors p_i_, a given site A, characterized by the descriptors p_i_(A) can be compared to another site B, characterized by the descriptors p_i_(B), through comparison of the single descriptors, respectively. Thus, site A will be ranked higher than site B, *i.e.*, B ≤ A, if at least one descriptor for A is higher than the corresponding descriptor for B and no descriptor for A is lower than the corresponding descriptor for B. If, on the other hand, p_i_(A) > p_i_(B) for descriptor i and p_j_(A) < p_j_(B) for descriptor j, A and B will be denoted incomparable. Obviously, if all descriptors for A are equal to the corresponding descriptors for B, *i.e.*, p_i_(B) = p_i_(A) for all i, the two sites will have identical rank and will be considered as equivalent, *i.e.*, A = B. In mathematical terms this can be expressed as

(3) $$B\;\leq \;A\Leftrightarrow p_{i}(B)\;\leq \;p_{i}(A)\;\text{for}\;\text{all}\;\text{i}$$

It further follows that if A ≥ B and B ≥ C then A ≥ C. If no rank can be established between A and B these sites are denoted as incomparable, *i.e.*, they cannot be assigned a mutual order. Therefore POR is an ideal tool to handle incommensurable attributes.

In partial order ranking – in contrast to standard multidimensional statistical analysis – neither any assumptions about linearity nor any assumptions about distribution properties are made. In this way the partial order ranking can be considered as a non-parametric method. Thus, there is no preference among the descriptors. However, due to the simple mathematics outlined above, it must be emphasized that the method a priori is rather sensitive to noise, since even minor fluctuations in the descriptor values may lead to non-comparability or reversed ordering.

A main point is that all descriptors have identical orientations, *i.e.*, “high” and “low”. As a consequence of this, it may be necessary to multiply some descriptors by −1 in order to achieve identical directions. As an example bioaccumulation and toxicity can be mentioned. In the case of bioaccumulation, the higher the number the higher a chemical substance tends to bioaccumulate and thus the more problematic the substance, whereas in the case of toxicity, the lower the Figure the more toxic the substance. Thus, in order to secure identical directions of the two descriptors, one of them, *e.g.*, the toxicity Figures, has to be multiplied by −1. Consequently, both in the case of bioaccumulation and in the case of toxicity higher Figures will now correspond to more problematic sites.

The graphical representation of the partial ordering is often given in a so-called Hasse diagram [55–58]. In practice the partial order rankings are performed using the WHasse software [58]. An alternative to the WHasse software is the DART (Decision Analysis by Ranking Techniques) that comprises different kinds of order ranking methods, roughly classified as total - and partial order ranking methods [59] or the PyHasse software currently being developed by R. Brüggemann [60].

#### 3.2.1. Linear extensions and ranking probabilities

The number of incomparable elements in the partial ordering constitutes a limitation in the attempt to rank, *e.g.*, a series of chemical substances based on their potential environmental or human health hazard. To some extent this problem can be remedied through the application of the so-called linear extensions of the partial order ranking [61,62]. A linear extension is a total order, where all comparabilities of the partial order are reproduced [54,55]. Due to the incomparabilties in the partial order ranking, a number of possible linear extensions correspond to one partial order. If all possible linear extensions are found, a ranking probability can be calculated, *i.e.*, based on the linear extensions the probability that a certain element has a certain absolute rank can be derived. If all possible linear extensions are found it is possible to calculate the averaged ranks of the single elements in a partially ordered set [63,64].

#### 3.2.2. Averaged ranks

Based on the linear extensions the averaged rank of the single elements can be established. The averaged rank is simply the averaged of the ranks in all the linear extensions. On this basis the most probable rank for each element can be obtained leading to the most probably linear rank of the elements studied.

The generation of the averaged rank of the single element in the Hasse diagram can be obtained through deriving a large number of randomly generated linear extensions [65–67]. The random linear extension approach allows in addition to the determination of the averaged ranks of the single elements also the determination of the ranking probability distribution of the single elements (cf. [14,15]).

Alternatively the generation of the averaged rank of the single sites in the Hasse diagram is obtained applying the simple relation recently reported by Brüggemann *et al* [68]. The simple relation can obtain the averaged rank of a specific element, c_i_.

(4) $$Rk_{av}(c_{i})\;=\;(N(c_{i})+1)-(S(c_{i})+1)\times (N(c_{i})+1)/(N(c_{i})+1-U(c_{i}))$$

where N(c_i_) is the number of elements in the diagram, S(c_i_) the number of successors, *i.e.*, comparable element located below, to c_i_ and U(c_i_) the number of elements being incomparable to c_i_ [68]. It is immediate seen that in the ranking according to eqn. 4 the lower the number the higher the levels. Thus, the highest level will be “1”. This is reversed compared to the original approach [68].

#### 3.2.3. Hierarchical POR

Based on the linear extensions the averaged rank of the single elements can be established. The averaged rank is simply the averaged of the ranks in all the linear extensions. On this basis the most probable rank for each element can be obtained leading to the most probably linear rank of the elements studied. These linear ranks can be regarded as meta-descriptors. If a series of such metadescriptors are generated from a set of partial order rankings they subsequently may constitute the basis for further ranking in a second stage, *i.e.*, a consecutive POR.

By this process the number of descriptors is significantly reduced and the ranking based on metadescriptors may, in contrast to a simultaneous inclusion of all original descriptors, lead to development of a robust model [69] that in principle will contain all information based on the original set of descriptors [16].

**Figure 7.**
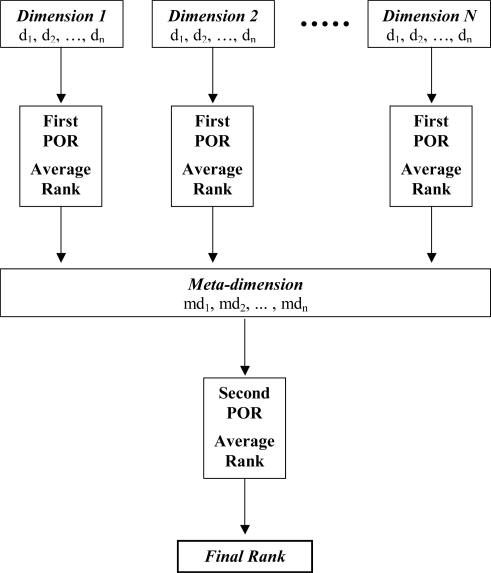
Graphical representation of the hierarchical partial order ranking [16].

Since the meta-descriptors, as the descriptors, are ordered with the highest rank being denoted “1”, the meta-descriptors must all be multiplied by −1 in order to make sure that the elements with the highest rank, *i.e.*, with the lowest attributed number, will be ranked in the top of the Hasse diagram as a result of the ranking based on the meta-descriptors. In Figure 7 a graphical representation of the HPOR approach is depicted.

### 3.3. Formal Concept Analysis (FCA)

Formal concept analysis (FCA) is a methodology to derive linkages between a set of objects, *e.g.*, chemicals, and a set of associated parameters, *e.g.*, the properties of these chemicals [17,18]. Thus, in short FCA can be as a system consisting of three parts, a context, or a triple (*C,P,L*), where *C* are the set of objects (chemicals) and *P* the set of parameters. *L* is the relation between the two sets *C* and *P*. Thus, if a chemical, *c*, belongs to the set *C* and *c* a parameter, *p*, belonging to the set *P*, (*c,p*) is said to belong to *L*.

The set of parameters that are associated with a given object, chemical, can be regarded as a set of binary, *i.e.*, on/off statements. Either the chemical has a given parameter, *e.g.* being carcinogenic, or not.

Typically a context will be seen as arranged in matrix form with the single objects as rows and the associated parameters as columns. Hence, an “X” in this table will indicate that a given object has the given parameter (on-status) whereas an empty space indicates that this parameter is not associated with the given object (off-status). Examples of contexts are given in Table XX and YY (*vide supra*).

For the studies referred to in this review the software ConExp [70] was applied to generate the lattice line diagrams as well as the implication sets and association rules.

#### 3.3.1. Line diagrams

The lattice line diagram consists of circles, lines and the names of all objects/chemicals (given in white boxes) and parameters of the context (given in grey boxes) where the circles represent the concepts. Blue filled upper semi-circle indicates that there is an attribute attached to this concept. Black filled lower semi-circle indicates that there is an object attached to this concept.

From the diagram the information of a context can be read as: a chemical (object), *c*, has a parameter (characteristic), *p*, attached only if there is an upward line from the circles with the label *c* to a circle with the label *p*.

## 4. Conclusions

In the present study the interplay between QSAR/QSPR and partial order ranking and formal concept analyses reviewed. It has been demonstrated that QSAR/QSPR models advantageously can be used to generate physico-chemical and ecotoxicological data (EPI Suite) as well as data to elucidate possible adverse human health effects (ADME/Tox Boxes and PASS). It has further been demonstrated, using residual rockets fuel, 1,1-dimethylhydrazine, and a series of its transformation products as an illustrative example that a further data treatment advantageously can be carried out applying partial order ranking (POR) methodologies as well as formal concept analysis (FCA). Whereas the partial order ranking methodologies lead to a prioritization of the studied chemicals simultaneous taking a multitude of parameters into account, the formal concept analysis leads to valuable information on possible links between the studied chemicals and the associated parameters. As such the combination QSAR/QSPR – POR – FCA constitutes a highly effective decision support tool.

## Figures and Tables

**Table 1.** Calculated and experimentally determined physico-chemical parameters for the investigated substances^a^ [5].

No	Log S*_W_* mg/L	Log *K_OW_*	Log *K_OC_*	Log *HLC* atm m^3^ mole^−1^	Log *VP* mmHg

1	1×10^6^ (1×10^6^)	−1.19	1.29	6.95×10^−8^	1.68×10^2^ (1.57×10^2^)
2	1×10^6^ (8.9×10^5^)	0.04 (0.16)	1.17	1.28×10^−4^ (1.04×10^−4^)	1.69×10^3^ (1.61×10^3^)
3	1×10^6^ (1.7×10^6^)	−0.17 (−0.38)	1.12	1.81×10^−5^ (1.77×10^−5^)	1.52×10^3^ (1.47×10^3^)
4	1×10^6^	0.69	1.03	1.96×10^8^	21.3
5	9.6×10^5^ (1×10^6^)	−0.64 (−0.57)	1.58	2.02×10^−6^ (1.82×10^−6^)	4.3 (2.70)
6	1×10^6^ (1×10^6^)	−0.93 (−1.01)	0.38	7.38×10^−8^ (7.39×10^−8^)	3.49 (3.87)
7	1×10^6^	−0.52	1.53	7.39×10^−7^	1.31×10^2^
8	4.5×10^5^	0.40	1.85	5.91×10^−5^	80.3
9	7.78×10^5^	0.68	1.58	4.45×10^−5^	3.30×10^2^
10	1×10^6^	−0.73	1.45	1.53×10^−7^	1.45×10^2^
11	4.77×10^5^ (1×10^6^)	−0.17 (−0.34)	0.18	6.78×10^−5^ (6.67×10^−5^)	9.10×10^2^ (9.02×10^2^)
12	1×10^6^	−1.70	0.65	3.08×10^−10^	0.14
13	1×10^6^	−0.44	1.00	1.52×10^−8^	7.12
14	3.02×10^4^ (4.8×10^5^)	0.23 (−1.38)	1.16	3.45×10^−6^	35.2 (7.51×10^3^)
15	3.1×10^5^ (1×10^6^)	−0.69 (−0.25)	0.43	2.42×10^−2^ (1.33×10^−4^)	7.32×10^2^ (7.42×10^2^)
16	2.0×10^5^	0.33	2.37	3.60×10^−5^	3.78
17	5.7×10^5^	−0.21	2.16	3.26×10^−5^	10.5
18	7.3×10^4^	0.61 (0.23)	1.20	7.88×10^−5^	11.5

^a^ Values given in parentheses are experimental values as provided by the database associated with the EPI Suite.

**Table 2.** Calculated persistence of the investigated structures in the environment [5].

No.	*BDP3^a^*	Ultimate biodegradation half life within	Fast Anaerobic biodegradation?	Residence half life in rivers^b^	Residence half life in lakes^b^

1	3.0664	Weeks	Yes	272 d	8.1 y
2	2.8137	Weeks	No	5.1 h	5.0 d
3	3.1240	Weeks	Yes	22.9 h	12.8 d
4	2.9425	Weeks	Yes	3.67 y	40.1 y
5	2.6503	Weeks to Months	Yes	11.6 d	130 d
6	2.9834	Weeks	No	282 d	8.4 y
7	3.0044	Weeks	Yes	31.0 d	340 d
8	3.0088	Weeks	No	10.1 h	7.9 d
9	3.0398	Weeks	Yes	12.0 h	8.4 d
10	3.0354	Weeks	Yes	137 d	4.1 y
11	3.1241	Weeks	Yes	6.5 h	5.3 d
12	3.0045	Weeks	Yes	204 y	2220 y
13	2.6761	Weeks to Months	No	4.0 y	44.0 y
14	3.1615	Weeks	No	6.7 d	6.7 d
15	3.1394	Weeks	Yes	2.8 h	3.1 d
16	2.9097	Weeks	No	17.0 h	11.2 d
17	3.0155	Weeks	Yes	17.3 h	11.1 d
18	3.0177	Weeks	No	7.7 h	6.6 d

^a^ BDP3:Biodegradation potential for ultimate biodegradation [34]

^b^ h: hours, d: days, y: years. Biodegradation not taken into account

**Table 3.** ECOSAR derived baseline and acute toxicity of the investigated compounds (values above 100 are rounded) [5].

No.	***LC50* (mg/L)**			*EC50* (mg/L)

	Fish^a^	Fish^b^	Daphnids^c^	Green algae

1	48500	5.9	6.2	0.53^d^
2	4050	290	16.8	16.1^b^
3	4700	300	17.0	15.1^b^
4	2160	1470	1450	830^b^
5	19800	1000	5200	39.8^b^
6	35000	30800	27000	14200^b^
7	18500	4.4	6.1	0.67^d^
8	2850	1.7	3.5	0.53^d^
9	1350	1.1	2.5	0.42^d^
10	23800	4.6	5.8	0.59^d^
11	4600	17.8	48.8	1820^b^
12	200000	14.4	12.2	0.88^d^
13	15100	850	45.5	37.0^b^
14	800	580	550	310^b^
15	8000	6775	6025	3225^b^
16	3700	2675	2550	1450^b^
17	9400	7350	6775	3725^b^
18	1800	1225	1200	690^b^

^a^ Baseline (non polar) toxicity (14 day’s test);

^b^ polar toxicity 96 hrs;

^c^ polar toxicity 48 hrs;

^d^ polar toxicity 144 hrs

**Table 4.** ADME results (n/a: calculations not available) [6].

No	Passive absorption (Human intestinal)^a^	Absorption rate constant (min^−1^)	*PPB*%^b^	Binding constant log *K_a_^HSA^*	*Vd*^c^ (L/kg)	P-Glycoprotein inhibitor	P-Glycoprotein substrate

1	94 (10/90)	0.012	14.71	1.70	0.94	0.003	0.031
2	99 (53/47)	0.019	13.96	1.80	2.00	0.002	0.002
3	99 (18/82)	0.018	11.74	1.65	2.29	0.004	0.014
4	100 (98/2)	0.044	17.27	2.38	1.13	0.003	0.008
5	100 (88/12)	0.022	7.25	2.19	1.01	0.003	0.006
6	94 (76/24)	0.012	3.84	2.00	0.96	0.004	0.007
7	100 (95/5)	0.031	17.49	2.24	1.22	0.003	0.010
8	100 (98/2)	0.050	26.73	2.41	1.24	0.009	0.006
9	100 (93/7)	0.031	19.85	2.22	1.14	0.005	0.005
10	93 (34/66)	0.011	20.17	1.81	1.22	0.004	0.039
11	100 (74/26)	0.022	5.67	2.10	1.03	0.004	0.006
12	89 (76/24)	0.009	7.33	2.05	0.94	0.004	0.011
13	99 (91/9)	0.019	22.82	2.14	1.02	0.009	0.006
14	n/a	n/a	n/a	n/a	n/a	n/a	n/a
15	99 (50/50)	0.021	3.87	2.02	0.99	0.005	0.005
16	100 (94/6)	0.027	12.51	2.47	1.04	0.005	0.009
17	99 (85/15)	0.017	8.90	2.33	1.01	0.003	0.008
18	100 (94/6)	0.032	12.64	2.49	1.14	0.005	0.009

^a^ Values correspond to maximum passive absorption. Values in parentheses denote the respective transcellular/paracellular contributions

^b^ Plasma Protein Bound fraction

^c^ Volume of distrution

**Table 5.** Predicted probabilities for the compounds to exhibit adverse organ specific health effects (n/a denotes that calculated values are not available) [6].

No	**Probability for adverse health effects^a^**					
	Blood	Cardiovascular	Gastrointestinal	Kidney	Liver	Lungs

1	0.57	0.40	0.65 T	0.28	0.48 T	0.34 T
2	0.44	0.34	0.80	0.20	0.18	0.27
3	0.20	0.31	0.26	0.11	0.20 T	0.20 T
4	0.79	0.07	0.92	0.57	0.85	0.74
5	0.76	0.06	0.97	0.75 T	0.93 T	0.71 T
6	0.27	0.12	0.65	0.14	0.05	0.40
7	0.52	0.33	0.83	0.19	0.10	0.17 T
8	0.63	0.06	0.84	0.31	0.05	0.75
9	0.32	0.08	0.90	0.42	0.07	0.72
10	0.53	0.64	0.66	0.14	0.29 T	0.29 T
11	0.19	0.08	0.25	0.09	0.04	0.04 T
12	0.48	0.14	0.71	0.15	0.28	0.42
13	0.47	0.21	0.89	0.18	0.12	0.47
14	n/a	N/a	n/a	n/a	n/a	n/a
15	0.10	0.08	0.81	0.09	0.05	0.27
16	0.14	0.02	0.46	0.03	0.06	0.04
17	0.12	0.02	0.46	0.07	0.02	0.05
18	0.08	0.02	0.36	0.04	0.02	0.05

^a^ T denotes that tumors have been found in experimental studies

**Table 6.** PASS predictions of selected biological activities^a^ [6].

No	Carcinogenic	Mutagenic	Teratogenic	Embryotoxic

1	0.955 (0.002)	0.762 (0.006)	0.689 (0.031)	0.672 (0.016)
2	0.619 (0.001)	NE	NE	0.527 (0.043)
3	NE	NE	0.563 (0.062)	NE
4	0.894 (0.003)	0.792 (0.005)	0.946 (0.006)	0.816(0.007)
5	0.980 (0.001)	0.969 (0.002)	0.952 (0.005)	0.866 (0.005)
6	0.951 (0.002)	NE	0.614 (0.048)	0.795 (0.009)
7	0.827 (0.006)	0.539 (0.010)	0.698 (0.030)	0.604 (0.026)
8	0.980 (0.002)	NE	NE	NE
9	0.683 (0.012)	NE	NE	NE
10	0.923 (0.006)	0.619 (0.007)	0.811 (0.012)	0.681 (0.015)
11	0.628 (0.011)	NE	NE	NE
12	0.897 (0.003)	0.524 (0.011)	0.530 (0.072)	NE
13	NE	NE	NE	NE
14	n/a	n/a	n/a	n/a
15	NE	NE	NE	NE
16	NE	NE	NE	NE
17	NE	NE	NE	NE
18	NE	NE	NE	NE

^a^ Values given are the calculated probability for the compounds to exhibit the effect (only values above 0.5 is given). Values in parentheses are the calculated probabilities for the compounds for not exhibiting the effect. NE indicates that if the compound exhibit the effect the probability will be below 0.5.

^b^ n/a: PASS results not available for this compound

**Table 7.** Averaged rank calculated according to eqn. 4 (na: calculations not available) [6].

No	*Rkav* According to ToxBoxes^a^	*Rkav* According to PASS^b^

1	6.0	2.8
2	6.8	9.7
3	13.5	9.7
4	1.1	2.8
5	1.2	1.0
6	11.5	3.0
7	8.0	5.1
8	2.6	3.6
9	4.0	10.1
10	6.0	2.6
11	16.9	11.3
12	5.4	6.0
13	4.9	17.0
14	na	na
15	10.8	17.0
16	15.0	17.0
17	15.6	17.0
18	16.8	17.0

^a^ *Rkav* based on GAS, LIV, LUN

^b^ *Rkav* based on CAR, MUT, TER, EMB

**Table 8.** Averaged rank calculated according to eqn. 4 Based on Tux Boxes and PASSS (HPOR approach) (n/a: calculations not available) [6].

No	*Rkav*

1	5.1
2	9.0
3	12.0
4	1.1
5	1.1
6	8.2
7	8.3
8	3.6
9	6.5
10	2.8
11	16.6
12	6.0
13	9.0
14	n/a
15	13.2
16	14.8
17	15.9
18	16.9

**Table 9.** Selected implication sets from the formal concept analysis of human health effects by 1,1-dimethyl hydrazine and its transformation products as derived by PASS [35].

No of compounds	If	Then

7	M > 50	C > 60T > 50
5	M > 60	C > 60T > 60E > 60
1	M > 90	C > 90T > 90E > 80
7	T > 60	C > 60E > 60
4	T > 80	C > 60M > 60E > 60
3	T > 90	C > 60E > 80
4	E > 70	C > 60T > 60
3	E > 80	C > 60M > 70T > 90

**Table 10.** Selected association rules from the formal concept analysis of human health effects by 1,1-dimethyl hydrazine and its transformation products as derived by PASS [35].

No of compounds	Pct	If	Then

7 / 8	88	C > 60T > 50	T > 60E > 60
7 / 8	88	C > 60T > 50	M> 50
7 / 8	88	C > 80	T > 50
6 / 7	86	C > 60T > 60E > 60	M > 50
6 / 7	86	C > 60T > 60E > 60	C > 80
6 / 7	86	C > 60 M > 50T > 50	C > 80
5 / 6	83	C > 60M > 50T > 60E > 60	M > 60
5 / 6	83	C > 60M > 50T > 60E > 60	C > 80
4 / 5	80	C > 60M > 60T > 60E > 60	T > 80
4 / 5	80	C > 60M > 50T > 60E > 60	M > 70
4 / 5	80	C > 60M > 50T > 60E > 60	C > 80
4 / 5	80	C > 90	T > 60 E > 60

## Appendix

**Appendix 1.** Context table for ecotoxicological effects by 1,1-dimethyl hydrazine and its transformation products as derived by EcoSAR^a^ [46].

No.	F1000	F100	F10	F5	F1	D1000	D100	D10	D5	D1	A1000	A100	A10	A5	A1
1	**X**	**X**	**X**			**X**	**X**	**X**			**X**	**X**	**X**	**X**	**X**
2	**X**					**X**	**X**				**X**	**X**			
3	**X**					**X**	**X**				**X**	**X**			
4											**X**				
5	**X**										**X**	**X**			
6															
7	**X**	**X**	**X**	**X**		**X**	**X**	**X**			**X**	**X**	**X**	**X**	**X**
8	**X**	**X**	**X**	**X**		**X**	**X**	**X**	**X**		**X**	**X**	**X**	**X**	**X**
9	**X**	**X**	**X**	**X**		**X**	**X**	**X**	**X**		**X**	**X**	**X**	**X**	**X**
10	**X**	**X**	**X**	**X**		**X**	**X**	**X**			**X**	**X**	**X**	**X**	**X**
11	**X**	**X**				**X**	**X**								
12	**X**	**X**				**X**	**X**				**X**	**X**	**X**	**X**	**X**
13	**X**					**X**	**X**				**X**	**X**			
14	**X**					**X**					**X**	**X**			
15															
16															
17															
18											**X**				

^a^ F1000, F100, F10, F5 and F1 denote EcoSAR derived toxicities towards fish being higher than 1,000, 100, 10, 5 and 1 mg/L, respectively. Analogously for D (daphnids) and A (algae).

## Appendix

**Appendix 2.** Context table for carcinogenic, mutagenic, teratogenic and embryotoxic action by 1,1-dimethylhydrazine and its transformation products as derived by PASS^a^ [35].

No.	C5	C6	C7	C8	C9	M5	M6	M7	M8	M9	T5	T6	T7	T8	T9	E5	E6	E7	E8	E9
1	**X**	**X**	**X**	**X**	**X**	**X**	**X**	**X**			**X**	**X**				**X**	**X**			
2	**X**	**X**				**X**	**X**	**X**			**X**	**X**	**X**	**X**	**X**	**X**	**X**	**X**	**X**	
3											**X**									
4	**X**	**X**	**X**	**X**		**X**	**X**	**X**			**X**	**X**	**X**	**X**	**X**	**X**	**X**	**X**	**X**	
5	**X**	**X**	**X**	**X**	**X**	**X**	**X**	**X**	**X**	**X**	**X**	**X**	**X**	**X**	**X**	**X**	**X**	**X**	**X**	
6	**X**	**X**	**X**	**X**	**X**						**X**	**X**				**X**	**X**	**X**		
7	**X**	**X**	**X**	**X**		**X**					**X**	**X**				**X**	**X**			
8	**X**	**X**	**X**	**X**	**X**															
9	**X**	**X**																		
10	**X**	**X**	**X**	**X**	**X**	**X**	**X**				**X**	**X**	**X**	**X**		**X**	**X**			
11	**X**	**X**																		
12	**X**	**X**	**X**	**X**		**X**					**X**									
13																				
15																				
16																				
17																				
18																				

^a^ C5, C6, C7, C8 and C9 denote PASS predicted probabilities for the compound being carcinogenic higher than 50, 60, 70, 80 and 90%, respectively. Analoguously for M (mutagenic), T (teratogenic) and E (embryotoxic).
